# Is fusion to the stable sagittal vertebra necessary to avoid distal junctional kyphosis in thoracic adolescent idiopathic scoliosis?

**DOI:** 10.1007/s43390-025-01183-z

**Published:** 2025-09-09

**Authors:** Lærke Ragborg, Martin Heegaard, Thomas Andersen, Rose-Marie Høi-Hansen, Martin Gehrchen, Benny Dahl, Søren Ohrt-Nissen

**Affiliations:** Spine Unit, Department of Orthopedic Surgery, Rigshospitalet, Inge Lehmanns Vej 6, 2100 Copenhagen, Denmark

**Keywords:** Adolescent idiopathic scoliosis, AIS, Distal junctional kyphosis, DJK, Stable sagittal vertebra, SSV, Fusion length

## Abstract

**Study design:**

This is a retrospective single-center study.

**Purpose:**

The purpose is to investigate the incidence of distal junctional kyphosis (DJK) when fused proximal to the stable sagittal vertebra (SSV) in adolescent idiopathic scoliosis (AIS) patients undergoing selective thoracic fusion.

**Methods:**

We retrospectively reviewed a consecutive cohort of surgically treated AIS patients with Lenke 1–2 A/B curves between 2011 and 2022 with a minimum of 2 years of follow-up. The SSV was defined as the vertebra bisected by the posterior sacral vertical line on long-standing sagittal radiographs. All patients underwent posterior pedicle screw instrumentation, and the decision of fusion level was at the surgeons’ discretion. Distal junctional kyphosis was defined as ≥10° angulation between the lower instrumented vertebra (LIV) and the vertebra below the LIV (LIV + 1). Patients were stratified into Fusion proximal of SSV (Prox-SSV) and fusion including SSV (Incl-SSV). Multivariable backward regression was performed to identify predictors for DJK.

**Results:**

A total of 196 patients were included, with 80 in the Prox-SSV group. The overall DJK rate was 3.6% (7/196), occurring in 6.3% (5/80) in the Prox-SSV group and 1.7% (2/116) in the Incl-SSV group, respectively (*p* = 0.125). Fusion proximal of SSV did not significantly increase DJK risk (Univariate OR 7.98, 95% CI 0.87–66.6; excluded in multivariable regression). Using SSV for LIV selection would extend the fusion by one level in 63.8%, two in 25.0%, and three in 11.2% of patients.

**Conclusion:**

The overall risk of DJK is small in thoracic curves and fusion proximal to the SSV did not significantly increase the risk of DJK. Standardized use of SSV as LIV would result in a substantial extension of the fusion area with questionable benefits to the patients.

## Introduction

Selection of fusion levels in the surgical treatment of adolescent idiopathic scoliosis (AIS) is complex, as motion preservation and sufficient construct length to avoid adding-on or distal junctional kyphosis (DJK) often are contradictory [1]. Traditionally, the primary focus has been on curve correction in the coronal plane, and the selection of the lowest instrumented vertebra (LIV) in thoracic curves is often guided by coronal landmarks like the last substantially touched vertebra (LSTV) or the last touched vertebra (LTV) [1–4]. Using the stable sagittal vertebra (SSV) has been proposed as a key sagittal parameter to consider when choosing fusion levels [5, 6]. Studies have suggested an elevated risk of distal junctional kyphosis (DJK) in cases where fusion is ending proximal to the SSV [5–8], and concurrently reporting an incidence of up to 19% depending on the curve type [9]. In cases where discordance between the LSTV and the SSV is present, the determination of fusion length and level selection remains largely reliant on the clinical judgment and preference of the operating surgeon. With limited literature on the topic, further evidence is needed to determine whether including the SSV in the fusion provides significant benefits.

This study aimed to investigate the overall rate of DJK, and the association between fusion proximal to the SSV and the risk of developing post-operative DJK in AIS patients undergoing selective thoracic fusion.

## Methods and materials

### Data collection

**Fig. 1:**
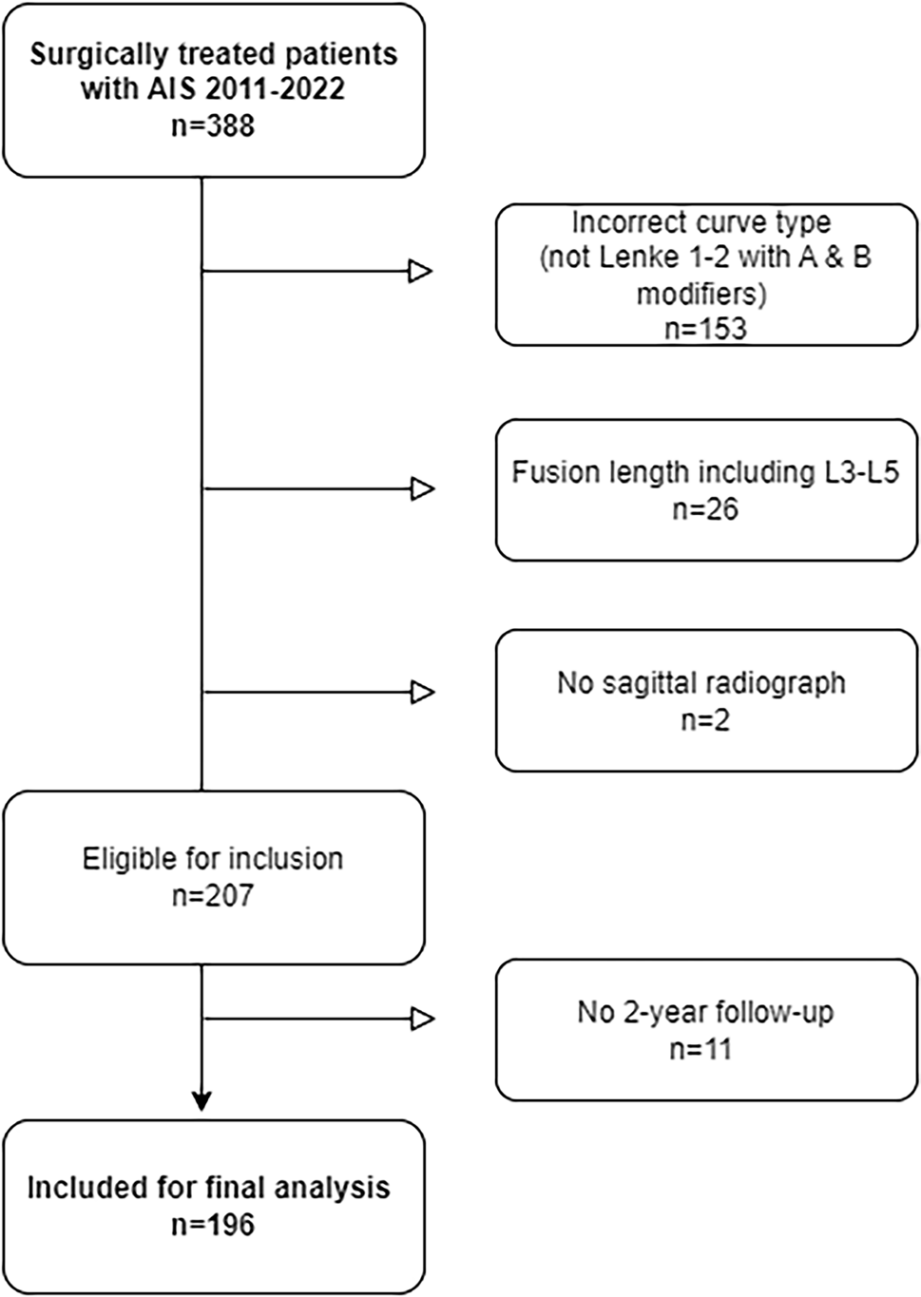
Inclusion flowchart

We retrospectively reviewed a consecutive cohort of AIS patients who underwent surgery at a single institution between 2011 and 2022. The inclusion criteria were: Lenke 1 or 2 curves with A or B lumbar modifiers, all-pedicle screw constructs with LIV at L2 or above, full-spine anterior–posterior and lateral radiographs including femoral heads preoperatively, postoperatively, and at 2 years postoperatively (Fig. 1). Demographics and data on revision procedures caused by DJK were collected from our electronic medical records.

### Radiographic parameters

Radiographic measurements were performed in the validated imaging software KEOPS (SMAIO, France [10]). These included major curve, secondary curve, LSTV, global kyphosis (maximum kyphosis angle), Δ global kyphosis, global lordosis (maximum lordotic angle), T10-L2 angle°, pelvic incidence (PI), pelvic tilt (PT), sacral slope (SS), DJK angle, and SSV. Distal junctional kyphosis was defined as a segmental kyphotic angulation between the superior endplate of LIV and the inferior endplate of LIV + 1 of ≥10°. The LSTV was defined as the most distal vertebra in the major curve touched by the center sacral vertical line (CSVL) in the coronal plane. The SSV was defined as the vertebra bisected by the posterior sacral vertical line (PSVL) [6]. Patients were grouped according to the position of the SSV relative to the LIV in a group fused proximal to the SSV (Prox-SSV) and a group including SSV in the fusion construct (Incl-SSV). In the Prox-SSV group, we collected the number of vertebras between LIV and SSV (ΔLIV-SSV) to determine loss of distal motion segments had they been fused to SSV.

### Surgical techniques

The surgical procedure used a standardized approach: partial facetectomies were performed at all planned fusion levels, with complete removal of the inferior facet, while the superior facet was left intact. Segmental uniplanar low-profile pedicle screws were used for fixation. Differential rod contouring, rod derotation, and direct vertebral rotation were applied. The convex rod was contoured to achieve the desired thoracic kyphosis. A high-stiffness construct with cobalt-chromium rods was used, targeting a screw density of 2.0.

### Statistics

Statistical calculations were performed in R, version 4.2.0 (R Development Core Team, Vienna, Austria 2020) [11]. Data was assessed for normality using histograms and Q-Q plots. Gaussian-distributed data were reported as means with standard deviations (SD) and compared using Student’s t-test. Non-gaussian distributed data were reported as medians with interquartile ranges [IQR] and compared with the Mann–Whitney U test. Counts (%) were compared using Fisher’s exact test. A univariable and stepwise backward multivariate logistic regression model including sex, age, pre-operative radiographic parameters, and the dichotomized variable “SSV included in fusion” to establish predictors of the risk of developing DJK was performed and reported with Odds Ratios (OR) with 95% Confidence intervals (95%CI).

A *p* value of <0.05 was considered statistically significant.

### Ethical considerations and approvals

The study was approved by the National Health and Medical Authority and the National Data Protection Agency (May 20, 2020: 31-1521-327; Oct 21, 2021: P-2021-779).

## Results

We included 196 patients. The mean age was 15.9 ± 2.2 years, and 85% (167/196) were females. Of these, 80 patients were fused proximal to the SSV (Prox-SSV), and 116 had a construct including the SSV (Incl-SSV). There was a small difference in BMI (21.8 ± 5.7 vs. 20.3 ± 2.8, *p* = 0.042) at baseline. Radiographic pre- and 2-year parameters and Lenke curve types are summarized in Tables 1 and 2.

**Table 1:** Pre- and post-operative radiographic parameters

Variables	Prox-SSV *n* = 80	Incl-SSV *n* = 116	*p* value
Major curve°, preoperative	59 ± 11	62 ± 13	**0.028**
Major curve°, 2 year	30 ± 11	28 ± 10	0.485
Secondary curve°, preoperative	36 ± 8	37 ± 10	0.305
Secondary curve°, 2 year	19 ± 10	19 ± 10	0.972
Global kyphosis°, preoperative	37 ± 11	33 ± 15	**0.017**
Global kyphosis°, 2 year	42 ± 10	38 ± 13	**0.021**
ΔGlobal kyphosis°	5 ± 11	5 ± 13	0.810
T10-L2°, preoperative	−4 ± 9	4 ± 10	**<0.001**
T10-L2°, 2 year	5 ± 10	1 ± 10	**0.003**
DJK angle°, preoperative	2 ± 5	1 ± 5	0.310
DJK angle°, 2 year	0 ± 5	−5 ± 5	**<0.001**
Global lordosis° preoperative	61 ± 11	62 ± 12	0.614
Global lordosis°, 2 year	61 ± 10	64 ± 9	0.071
Pelvic incidence°	46 ± 13	50 ± 12	**0.012**
Pelvic tilt°, preoperative	8 ± 10	8 ± 9	0.880
Pelvic tilt°, 2 year-	7 ± 8	8 ± 7	0.138
Sacral slope°, preoperative	37 ± 9	42 ± 10	**0.001**
Sacral slope°, 2 year	38 ± 9	42 ± 8	**0.002**
Sagittal vertical axis (mm), preoperative	−35 ± 35	−16 ± 32	**<0.001**
Sagittal vertical axis (mm), 2 year	−18 ± 39	−10 ± 35	0.160
Discordant LSTV and SSV	62 (78%)	95 (82%)	0.472

*Prox-SSV* patients fused proximal of SSV, *I-SSV* patients fused including SSV. Bold indicates a p-value <0.05

**Table 2:** Distribution of Lenke curve types

Lenke type	Prox-SSV *n* = 80	Incl-SSV *n* = 116	*p*-value
1A	51 (63.8%)	72 (62.0%)	
1B	18 (22.5%)	11 (10.5%)	
2A	10 (12.5%)	29 (25.0%)	
2B	1 (1.2%)	4 (3.5%)	**0.021**

The overall DJK rate was 3.6% (7/196). Of these, 6.3% (5/80) were in the Prox-SSV group, and 1.7% (2/116) in the I-SSV (*p*-value = 0.125). None of the patients underwent revision surgery due to DJK. Five of seven who experienced DJK had a Lenke 1A curve, one 1B curve, and one 2A curve. In the Prox-SSV group, the ΔLIV-SSV was one level in 51 (63.8%), two levels in 20 (25%), and three levels in 9 (11.2%) of patients, respectively.

Using backward multivariable logistic regression, we found a significantly decreased OR for DJK per increase in pre-operative T10-L2° of 0.69 (95% CI 0.52–0.91) and Major Curve° of 0.83 (95% CI 0.70–0.99). An increased OR for DJK was found per increase in preoperative DJK angle of 1.40 (95% CI 1.01–1.95). No other variables including SSV were significantly associated with DJK (Table 3).

**Table 3:** Logistic regression analysis—odds ratio of DJK 2 years postoperatively

Variable	Univariable OR (95% CI)	Multivariable OR (95% CI)_a_
Major coronal curve°, preoperative	0.90 (0.81–1.00)	**0.83 (0.70–0.99)**
Sex, male	1.12 (0.13–9.97)	–
Age, preoperative	1.55 (1.10–2.20)	–
Kyphosis°, preoperative	1.03 (0.96–1.09)	–
Lordosis°, preoperative	0.95 (0.88–1.03)	–
T10-L2°, preoperative	0.82 (0.73–0.92)	**0.69 (0.52–0.91)**
DJK angle°, preoperative	1.16 (1.01–1.33)	**1.40 (1.01–1.95)**
PI°, preoperative	0.95 (0.88–1.02)	–
PT°, preoperative	1.03 (0.95–1.11)	–
SS°, preoperative	0.89 (0.82–0.97)	–
SVA (mm), preoperative	0.99 (0.96–1.02)	–
SSV not included in fusion	7.63 (0.87–66.60)	–

*OR* odds ratio, *(95%CI)* 95% confidence interval.  Bold indicates a p-value <0.05

^a^Stepwise backward multiple logistic regression

## Discussion

The present study aimed to investigate the relationship between the development of post-operative DJK and fusion constructs with or without the inclusion of the SSV. We found a low overall rate of DJK, with no significantly increased risk of developing post-operative DJK in the Prox-SSV group.

While the rate of DJK is often presented in studies on AIS surgery, only a few have investigated the risk and preventative factors associated with DJK For the same reasons, the consequence of post-operative DJK in AIS patients is based on results from adult spinal deformity surgery, which may not accurately reflect the symptoms seen in the adolescent population, namely progressive loss of lordosis and disc height, spinal instability, and stenosis [12, 13]. However, the segmental deformity may contribute to the development of pain and adjacent segment disease over time. Consequently, accurate preoperative surgical planning is essential to mitigate the risk of DJK. In this current study, our DJK rates were lower than the previous reports (4% vs 6–15%) [8, 14–16], with none of the patients undergoing revision surgery for DJK. There might be several reasons for the range in the reported DJK incidence. Firstly, Ball et al. found high variability in SSV levels dependent on positional changes influenced by, e.g., hand placement, resulting in SSV changes in 44% of their cases based on serial radiographs [17] and thus concluded that SSV for LIV selection should be used cautiously. Therefore, the results presented in this study may vary from previously presented data, purely based on how the radiographs were performed. Moreover, sagittal malalignment has been associated with an increased risk of proximal and distal junctional failure, especially pre- and post-operative abnormal kyphosis [15, 18]. Mac-Thiong et al. described the kyphosis magnitude in an adolescent background population of 44± 11° [19], similar to the values seen in this study. Moreover, a similar kyphosis correction was seen in both groups, despite differences in the overall global kyphosis. This may explain why the incidence in this cohort is lower than reported elsewhere. As segmental kyphosis measurements vary greatly between studies (e.g. T2–T12, T5–T12, and global kyphosis), a direct comparison of the obtained kyphosis is challenging and may further explain the variability reported. Variability in segmental kyphosis measurement (e.g. T2–T12, T5–T12, global kyphosis and T10-L2) makes direct comparison between studies difficult and may contribute to the inconsistent DJK rates reported. This study found significant differences in pre- and postoperative T10–L2 angles between groups, with an associated decreased odds ratio (OR) for postoperative DJK. Notably, the inclusion of SSV in the fusion construct did not predict DJK. Additionally, larger preoperative DJK angles were predictive of the presence of postoperative DJK (Tables 1 and 2), which were in concordance with the findings by Lowe et al. [14]. These findings suggest that sagittal thoracolumbar alignment, rather than SSV inclusion, may be one of the primary drivers of DJK development. Our results indicate that a lordosis or hypokyphosis at the thoracolumbar junction (low T10-L2 angle or low DJK angle) predisposes to DJK when the global kyphosis is corrected during surgery, theoretically, due to a local stress riser at the LIV junction. However, this association was not the primary aim of the study and future investigations should focus on the relevance of local sagittal alignment in DJK risk assessment.

Several of the previous studies found an increased risk of DJK when LIV was proximal to the SSV [5, 6, 8]. Furthermore, fusion constructs extending into the lumbar region have been shown to reduce the likelihood of junctional failure [20]. It should be noted that several of these studies included type C modifiers and even structural double curves undergoing selective fusion. Wang et al. found a 6.6% DJK rate only occurring in the Prox-SSV group. The results showed an overall loss of thoracic kyphosis postoperatively, contrary to our study, which may explain the difference seen [5]. Yang et al. found the risk of DJK to be 17% when fusion was proximal to SSV (24 patients in this group). The study found no other significant predictors of DJK [6]. Unfortunately, postoperative radiographic parameters were not included, which complicates the comparison of the cohorts.

**Fig. 2:**
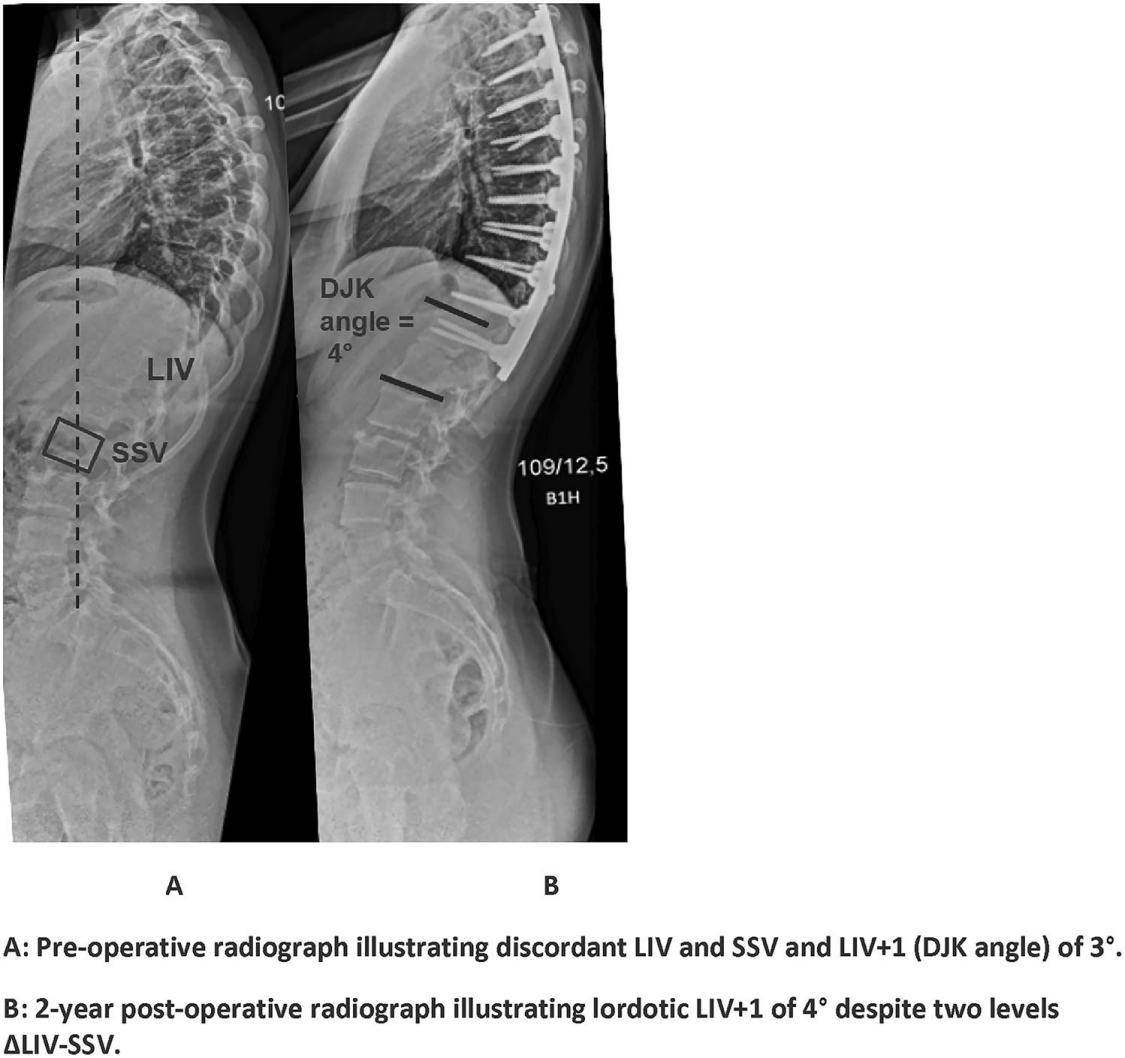
Pre- and post-operative radiograph of a Prox-SSV patient. **A** Pre-operative radiograph illustrating discordant LIV and SSV and LIV + 1 (DJK angle) of 3°. **B** 2-year post-operative radiograph illustrating lordotic LIV + 1 of 4° despite two levels ΔLIV-SSV

Standardizing SSV as the LIV choice will inevitably lead to longer instrumentations, impairing lumbar mobility [21, 22]. Therefore, the potential loss of distal motion segments should be weighed against the relatively low risk of DJK. Long-term studies indicate a correlation between fusion length, spinal mobility [22], and negatively impaired health-related quality of life (HRQOL), mainly explained by decreased function, mobility, and higher pain levels than in shorter fusions [23]. In this study, using SSV for LIV selection would have resulted in 80 patients (Prox-SSV group) having at least one extra level fused, and 35% of these would need ≥2 levels fused (Fig. 2). The LIV selection should therefore be based on both coronal and sagittal evaluation by the physician and shared decision-making with the patient, considering current and future functioning against the risk of DJK.

There are some limitations to this study. Due to the retrospective design, some inherent bias may be present. However, as we included a consecutive cohort of surgically treated patients, the risk of selection bias has been minimized. As the data were collected from medical records, the reasoning for LIV selection in the respective cases was unknown. The stable sagittal vertebra has not traditionally been considered in AIS fusion selection at our center and should pose a bias. If a thoracolumbar kyphosis is present fusion may be extended to include that, which could introduce a bias in our results. We did note a small difference in preoperative kyphosis between the groups, which was maintained postoperatively (Table 1). As we focused on Lenke 1 and 2 curves, our results may not be generalizable to other curve types and should be interpreted with this limitation in mind. As patient-reported outcome measures were not collected as a standard, we were unable to report on the influence of LIV selection, fusion length, and DJK on HRQOL. Whilst DJK is mainly known to impact HRQOL in adults negatively, additional data concerning the potential influence in adolescents would have provided valuable information for future decision-making. Finally, the previously reported variability in SSV selection may impact the findings presented and should be considered when comparing these results with those of other studies.

## Conclusion

The overall risk of DJK is small after selective thoracic fusion and fusion proximal to the SSV did not significantly increase the risk of DJK. Standardized use of SSV as LIV would result in a substantial extension of the fusion area with questionable benefits to the patients.

## Data Availability

The data that suppor the findings of this study are available upon reasonable request. The data is not publicly available due to privacy reasons.
